# Dietary Strategies to Mitigate Alzheimer’s Disease: Insights into Antioxidant Vitamin Intake and Supplementation with Microbiota–Gut–Brain Axis Cross-Talk

**DOI:** 10.3390/antiox13121504

**Published:** 2024-12-10

**Authors:** Wan Zurinah Wan Ngah, Hajar Fauzan Ahmad, Sheril June Ankasha, Suzana Makpol, Ikuo Tooyama

**Affiliations:** 1 Medical Innovation Research Center, Shiga University of Medical Science, Otsu 520-2192, Shiga, Japan; wanzurinah@ppukm.ukm.edu.my; 2 Department of Industrial Biotechnology, Faculty of Industrial Sciences and Technology, Universiti Malaysia Pahang Al-Sultan Abdullah, Gambang 26300, Pahang, Malaysia; fauzanahmad@umpsa.edu.my; 3 Unisza Science and Medicine Foundation Centre, Universiti Sultan Zainal Abidin, Gong Badak Campus, Kuala Nerus 21300, Terengganu, Malaysia; ankasha88@gmail.com; 4 Department of Biochemistry, Faculty of Medicine, Universiti Kebangsaan Malaysia, Bandar Tun Razak, Cheras, Kuala Lumpur 56000, Malaysia; suzanamakpol@ukm.edu.my

**Keywords:** Alzheimer’s disease, tau, amyloid-beta, gut microbiota, gut–brain axis, probiotic, antioxidant vitamins

## Abstract

Alzheimer’s disease (AD), which is characterized by deterioration in cognitive function and neuronal death, is the most prevalent age-related progressive neurodegenerative disease. Clinical and experimental research has revealed that gut microbiota dysbiosis may be present in AD patients. The changed gut microbiota affects brain function and behavior through several mechanisms, including tau phosphorylation and increased amyloid deposits, neuroinflammation, metabolic abnormalities, and persistent oxidative stress. The lack of effective treatments to halt or reverse the progression of this disease has prompted a search for non-pharmaceutical tools. Modulation of the gut microbiota may be a promising strategy in this regard. This review aims to determine whether specific dietary interventions, particularly antioxidant vitamins, either obtained from the diet or as supplements, may support the formation of beneficial microbiota in order to prevent AD development by contributing to the systemic reduction of chronic inflammation or by acting locally in the gut. Understanding their roles would be beneficial as it may have the potential to be used as a future therapy option for AD patients.

## 1. Introduction

Alzheimer’s Disease (AD) and dementia are debilitating diseases and affect a total of 55 million people worldwide, with AD accounting for 60–70% of all dementias. In developed countries, such as the United States, the prevalence rate of AD is 6.7 million people over the age of 65, and it is projected to increase to 12.7 million by 2050 [1]. In the European Union, more than 7.5 million people were affected by AD in 2019, which was a surprising reduction from the previously estimated value of 8.7 million [2]. However, these numbers are rising, correlating with the increasing aging population worldwide. While the number of AD patients in developing countries in Asia, Africa, and Latin America is lower compared to developed countries, the global aging population is steadily increasing. Global life expectancy at birth has been reported to increase from 66.8 years in 2000 to 73.3 years in 2019 [3]. More significantly, the world’s aging population is projected to grow, with the percentage of the population over 65 years old expected to increase from the current 10% to 16% in 2050 and 24% by 2100 [4]. This resulted in a substantial increase in AD.

Current treatment for AD includes cholinesterase inhibitors and an antagonist of the N-methyl-D-aspartate receptor, which manages dementia symptoms but does not halt or reverse the progression of the disease. The search for AD treatment continues, with clinical trials focusing on anti-amyloid β therapy showing limited success, despite the general acceptance of the involvement of amyloid β (Aβ) and tau proteins in AD pathology [5]. Studies continue to attempt to comprehend the mechanisms involved in AD development. Increasing evidence suggests the involvement of other cells, such as microglial cells, in maintaining brain health. This observation likely stems from the fact that centenarians remained cognitively healthy despite having brains populated with Aβ [6]. The mechanism involved in the development of AD encompasses the entire brain, including microglia–neuron interactions and the gut–brain axis, both of which contribute to overall brain health [7,8]. The gut–brain axis’s presence underscores the microbiota’s role in the development of AD, particularly with increasing evidence indicating differences between the gut microbiota of healthy individuals and AD patients [9].

Diet is reported to shape the composition of the gut microbiota, and the gut and its metabolites play a role in the development of AD [9]. Components of the diet that influence the type of gut microbiota include fiber from vegetables, which supports healthy microbiota. However, despite health recommendations by the USDA and governments, statistics revealed that only 10% and 26% of the population achieve the recommended 5–7 servings per day of fruits and vegetables in developed countries such as the USA and UK, respectively [10,11]. Meeting these recommendations may pose an even greater challenge for the elderly, who are at the highest risk of developing AD. Therefore, exploring the potential use of supplements to aid in maintaining a healthy gut microbiota, especially in the elderly or those experiencing aging, who are particularly susceptible to AD, is worth investigating. Various antioxidant vitamins and diet components have been reported to be protective and delay the development of AD through reduced inflammation, a factor widely accepted to be involved in AD pathogenesis [12,13], reduced pro-oxidants in the brain [14], and reversed AD [15]. Investigating whether antioxidant vitamins reduce inflammation and influence the gut microbiota would be intriguing. This review aims to explore whether antioxidant vitamins, either obtained from the diet or as supplements, can support the growth of beneficial microbiota and prevent AD development through the reduction of chronic inflammation either systemically or in situ in the gut, in either intestinal mucosal cells or the lumen. The gut microbiota interacts with the central nervous system (CNS) across the microbiota–gut–brain axis, involving microbial components, metabolic products, and neural stimulation.

## 2. Gut Microbiota and AD

Microbial communities associated with humans encompass bacteria, fungi, protists, archaea, and viruses, commonly referred to as the bacteriome, mycobiome, protistome, archaeaome, and virome. These microorganisms inhabit and reside on and within the human body [16]. Throughout every phase of life, from birth through to death and decomposition, microbial communities serve as a constantly evolving element within the body [17]. Delving into the inherent and triggered alterations in our microbiota holds the promise to fundamentally transform our comprehension of human biology during healthy and disease states [18,19,20]. While the reasons behind the prevalence of specific microorganisms at different ages remain largely unclear, research on the human microbiota has shed light on a sequence of changes that microbial communities undergo from birth to the end of life. The gut microbiota composition evolves across the different life stages. It is relatively sterile in infancy and becomes dominated by *Bifidobacteria* and lactobacilli, influenced by factors such as delivery mode and feeding [21]. Childhood and adolescence witness a growing and diversifying microbiota [22,23]. It becomes relatively stable in adulthood, reflecting long-term dietary habits [24,25]. However, old age often brings a less diverse microbiome [26], with reduced beneficial bacteria and increased potential pathogens influenced by factors such as age-related dietary changes and health conditions [27,28]. These variations underscore the importance of a healthy gut microbiota at every stage of life for overall well-being.

The composition of the gut microbiota is strongly influenced by aging, favoring the development of pro-inflammatory bacteria at the expense of anti-inflammatory bacteria. Studies have shown that the gut microbiota of older individuals differs from that of younger adults [29,30]. These changes occur gradually with time and can be influenced by factors such as diet, exercise, medication, and cohabitation patterns [31,32,33,34]. Loss of diversity in the core microbiota groups is associated with increased frailty [27]. The changes in the gut microbiota that occur with aging can be associated with diet and the reduced abundance of certain taxa [35]. While gut dysbiosis has conventionally been associated with a higher susceptibility to infections, there is a growing awareness that disruptions in the microbial community structure of the intestines, which are linked to undesirable inflammatory reactions, play a role in various diseases affecting multiple organ systems. Additionally, it is becoming increasingly evident that the relationship between gut dysbiosis and age-related illnesses might be rooted in how the gut microbiota communicates with the intestinal mucosa and the overall immune system, such as *Listeria monocytogenes* infection and age-related atrial fibrillation [36,37,38], leading to frailty. This leads to localized systemic inflammation, increased intestinal permeability, a dysfunctional blood–brain barrier, and ultimately neuroinflammation [39]. Recent evidence has indicated that the fecal matter transplantation (FMT) of gut microbiota from individuals with AD to rats has specific effects on the gut microbiota and intestinal epithelium. These alterations include a higher abundance of Bacteroidetes, which consists of pro-inflammatory species, and a lower abundance of the phyla Firmicutes and Verruocomicrobiota, which produce beneficial metabolites. This also leads to impairments in behaviors related to memory and mood [40]. These findings suggest that targeting the gut microbiota could potentially be a therapeutic approach for AD.

Aβ is a peptide naturally synthesized in the body, with the brain being its primary source. It is a normal by-product of the processing of a larger protein called the amyloid precursor protein [41]. In healthy individuals, Aβ is typically broken down and cleared from the brain [42]. However, in some circumstances, this process can become disrupted, leading to the accumulation of Aβ in the brain [43,44]. Its build-up is a distinctive characteristic observed in various neurodegenerative conditions, notably AD. In AD, Aβ aggregates to form plaques, which are toxic to neurons and disrupt normal brain function [45]. These Aβ plaques are thought to trigger a cascade of events, including inflammation, oxidative stress, and neuronal damage, ultimately leading to cognitive decline and the characteristic symptoms of the disease [46,47,48]. A growing body of evidence indicates that the microbiota–gut–brain axis not only influences brain cognition and psychiatric symptoms but also plays a role in the development of neurodegenerative diseases such as AD [49]. It has been reported that alterations in the diversity and makeup of the gut microbiota and the presence of dysbiosis are linked to the onset of brain disorders such as neurodegenerative diseases [50]. These connections operate through diverse mechanisms, including the promotion of inflammation, heightened oxidative stress, and disruptions in energy metabolism [51]. Additionally, some studies propose that specific gut bacteria such as *Pseudomonas*, *Salmonella*, and *Escherichia coli* may influence proteins such as Aβ and tau, which are associated with the development of neurodegenerative conditions [51,52,53]. The fact that amyloid is a significant secretory product of microorganisms and may have a sizable impact on the pathophysiology of the human CNS is often underappreciated [54]. Various microbiota species, including fungi and bacteria, secrete amyloid proteins. For instance, the association between amyloids and fungal surface structures and the recent discovery of amyloidogenic fungal proteins and widespread mycoses in the blood of AD patients point to a potential link between chronic fungal infections and an increased risk of AD [55,56]. Here, we have compiled studies conducted worldwide over the past five years related to AD, focusing on the identification of gut microbiota among human populations and diet intervention on animal models. In general, the idea that the gut microbiota plays a role in the onset of AD is supported by research on both humans and animals. The general patterns of dysbiosis, which are marked by a decrease in helpful microorganisms and an increase in harmful ones, are similar in humans and animals, despite certain species-specific variations in microbial composition. The findings are presented in Table 1 and Table 2, respectively.

### Mechanism of Functional Bacterial Amyloid Protein Production from Microorganisms

In 2002, the ground-breaking discovery of amyloid proteins produced by bacteria unveiled new insights into AD. The study described that curli operons in *Escherichia coli* are responsible for the production of curli fibers [81]. Notably, the major structural subunit of CsgA serves as a common secretion component of extracellular amyloids that play a pivotal role in facilitating surface attachment, adhesion, biofilm formation, and shielding against host defenses [54]. Extracellular polymeric amyloids and other lipoproteins, found in various structural configurations, comprise the complex matrix that forms a biofilm. It is interesting to note that the extracellular 17.7 kDa CsgA amyloid precursor contains a PAMP that, similar to the A-42 peptide, is recognized by the TLR2 receptor of the human immune system [81]. Subsequently, it has been demonstrated that several organisms residing within the human body possess the capability to produce amyloids, such as *Streptococcus*, *Staphylococcus*, *Salmonella*, *Mycobacterium*, *Klebsiella*, *Citrobacter*, and *Bacillus* species [81]. Other than curli produced by *Escherichia coli* and *Salmonella* spp., functional amyloids used by microbes to encourage bacterial interactions include chaplins made by *Streptomyces* spp. and TasA fibers made by *Bacillus subtilis* [81]. While bacterial amyloids serve as adaptive structures and provide defenses to bacterial communities by resisting degradation caused by viruses and other agents, it is noteworthy that bacterial amyloids have been associated with both infections and autoimmune reactions [81]. Hence, this suggests that bacterial amyloids may trigger the aggregation of neuronal proteins such as Aβ, alpha-synuclein, tau, and others, initiating a prion-like propagation process [82]. The sheer abundance and diverse array of gut microbiota and their ability to generate substantial amounts of amyloids suggest that human physiology could potentially face a considerable systemic amyloid load [81].

Initially, during amyloid production, the special characteristics of these microbial proteins enable them to take on amyloid-like conformations [83]. Importantly, these proteins are similar to their human counterparts implicated in amyloid formation in that they have areas that are prone to misfolding and aggregation [84]. Misfolding and nucleation are two further steps on the path to amyloid formation [85]. Microbial amyloidogenic proteins experience misfolding, which results in a conformational shift that exposes amyloidogenic areas, just like in human amyloidogenesis [86]. In the first phases of aggregation, these areas play a crucial role. The nucleation stage, in which a few misfolded proteins gather together to form a nucleus or seed that acts as the cornerstone for continued growth, is a crucial turning point [87]. The next critical phase in this process is the development of fibrils [88]. Once the nucleus has been formed, it serves as a template, drawing in and lining up more misfolded proteins [85]. This recruiting mechanism facilitates the elongation of the amyloid fibril. It is significant because the expanding fibril continues to draw in additional proteins with comparable structural characteristics, strengthening its structure [89]. As the amyloid fibril expands, its proteins adopt a specific conformation known as the cross-β sheet structure [90]. This distinct arrangement involves the alignment of β-strands perpendicular to the fibril axis, creating a characteristic repeating pattern [91,92]. The stability and propagation of these microbial amyloid fibrils are notable features. These fibrils are remarkably stable and resistant to degradation by standard protease enzymes. This stability contributes to their environmental persistence and potential to engage with host organisms [93].

How the microbial amyloids fare will depend on how they operate. For the bacteria that produce them, these amyloids may have a variety of uses [94]. Contributing to the development of biofilms, promoting microbial adherence to surfaces, offering defense against environmental stressors, including desiccation, and enabling interactions with host organisms are a few examples of potential functions [95]. This is particularly notable during the aging process, when the epithelial lining of the gastrointestinal tract and the blood–brain barrier undergo significant restructuring, potentially increasing their permeability [96,97]. The intestinal microbiota represents a dynamically evolving ecosystem that poses a significant potential threat, necessitating continuous vigilance. This surveillance on the epithelial surface entails the sampling of intestinal contents by epithelial microfold M and dendritic cells, which transport antigens to immune cells located in Peyer’s patches and other parts of the intestinal lymphoid system [98]. This hematogenous stimulation of the innate immune system might lead to an enhanced response to neuronal amyloids in the brain. Through the neural connections of enteroendocrine cells and other epithelial cells, microbial amyloids could also stimulate the production of neuronal amyloids, such as alpha-synuclein [99]. Exposure to microbial amyloids could potentially increase the accumulation of neuronal amyloids by upregulating expression and promoting cross-seeding, resulting in the misfolding of neuronal proteins in the brain [100].

## 3. Strategies That Support Healthy Microbiota for Brain Health

Increasing evidence highlights the significance of gut microbiota in individuals with AD, emphasizing the importance of maintaining a healthy microbiota for overall well-being. The microbiota contributes enzymes that are not encoded by the human genome, facilitating the breakdown of complex substances such as polysaccharides and polyphenols, as well as the synthesis of essential vitamins. A healthy microbial environment produces short-chain fatty acids (SCFAs), including acetate, propionate, and butyrate, which play a crucial role in maintaining the integrity of the epithelial barrier, modulating the immune system, and providing protection against infections [101]. Pathogenic and non-pathogenic bacteria, along with substances generated by the host (such as mucins, anti-microbial peptides, and immunoglobulins), engage in symbiotic interactions to uphold a healthy gut microbiome. This symbiosis is often characterized by a rich diversity of bacterial populations [102]. It is possible that dysbiosis affects the intestinal environment, which negatively impacts the brain through the gut–brain axis. The composition of gut microbiota, in general, can be modulated by various factors, including diet, nutritional intervention, caloric restriction or fasting, and the administration of prebiotics, probiotics, or psychobiotics [103,104,105].

### 3.1. Caloric Restriction or Fasting

Research on older people has demonstrated that calorie restriction (CR) has neuroprotective benefits on memory [106]. Research in mice has shown that CR diets improved hippocampus biology by affecting gene expression and nutrient-sensing pathways, with links to an increase in dendritic spines in the dentate gyrus and cognitive protection [107]. According to Zhang et al. (2013), in terms of gut microbiota, CR facilitates the development of healthy gut microbiota by lowering the intestinal antigen burden and significantly affecting the succession of gut microbiota during aging, with favorable impacts on the host’s health [108]. Nevertheless, in comparison to exercise, CR showed a more prominent impact on the overall architecture of the gut microbiota, particularly with unique microbiota clusters at both mid-life and late-life ages.

Intermittent fasting (IF) has also been demonstrated to improve neuropathological features and cognitive abnormalities in AD, where it leads to ketogenic metabolism and increases β-hydroxybutyrate levels to protect against Aβ toxicity [109,110]. In addition, it protects hippocampal murine neurons [111,112] and lowers amyloid deposition and tau pathology, hence improving learning and memory skills [113]. IF has been shown to have a direct impact on the gut microbiota. Based on previous studies in mice, fasting increased the level of 3-hydroxybutyric acid and 4-hydroxybutyric acid in the intestine, which may be related to changes in gut microbiota, with an increase of Firmicutes but a decreased abundance of Bacteroides [114]. A study found that IF reduced cognitive impairments and insulin resistance, with an increase in *Lactobacillus* and the butyrate producer *Odoribacter* [115].

### 3.2. Diet and Nutritional Intervention

Food has been shown to directly impact many of the fundamental characteristics of AD development, such as amyloid genesis, oxidative stress, and inflammation [116]. There has been limited exploration into the potential influence of dietary choices on AD progression. One of the healthiest dietary patterns globally is the Mediterranean (MED) diet, which underscores the consumption of non-refined grains, vegetables, fruits, whole grains, nuts, legumes, white meat, shellfish, fish, eggs, and dairy products while minimizing the intake of processed foods and red meat. This diet has been linked to enhanced cognitive function and a lower risk of developing dementia by reducing inflammation and oxidative stress and enhancing neurogenesis and neural connection [116,117,118]. Ghosh et al. (2020) discovered that the MED diet is helpful in changing the gut microbiota, lowering frailty, and increasing cognitive performance [119]. These microbiota changes in the MED diet intervention group are predominantly attributed to increased fiber, vitamins (C, B6, B9, and thiamine), and minerals (Cu, K, Fe, Mn, and Mg). In contrast, changes in the control group are primarily associated with increased fat intake, including saturated fats and monounsaturated fatty acids. Consequently, the MED diet has been identified as one of the top five modifiable risk factors for AD and cognitive decline.

The main component of a MED diet is a high intake of fermentable dietary fibers, also referred to as soluble fibers or prebiotic fibers. These fibers are linked to abundant good fiber-fermenting bacteria in the colon, which increase SCFA levels in the gut and bloodstream through fermentation [120]. For example, β-glucans, soluble fibers present in fungus, yeast, and cereal grains such as oats and barley, have been demonstrated to change microbiota and cognition in various rodent models. Memory improvements were observed in Aβ1-42-induced adult male C57BL/6J mice supplemented with yeast-derived β-glucan for 4 weeks, which correlated with changes in beneficial and inflammatory-related microbiota, with a relatively increased Bacteroidetes abundance and a decreased abundance of *Firmicutes*. In addition, there were improvements in brain insulin resistance and neuroinflammation and increased hippocampus SCFAs [121]. In another study, oat-derived β-glucan supplementation (7%) added to an HFD (55% kcal by fat) for 15 weeks prevented recognition memory deficits in adult male C57Bl/6J mice and reversed the microbiome changes, with increased *Bacteroides* and decreased Proteobacteria abundance, seen with the HFD alone [122]. Similar effects were also shown in a previous study where oat fiber improved spatial learning and memory deficits by improving microbiome diversity and boosting Actinobacteria while decreasing *Rikenellaceae*. In the distal colon, oat fiber additionally boosted the expression of SCFA receptors and tight junction proteins. In barley, which is a grain high in β-glucans, comparable results were discovered in a mouse model of age-related cognitive decline, where it was demonstrated to reduce cognitive deficits and change gut flora, such as *Bacteroides* and *Prevotella* [123].

Another important component of the MED diet is omega-3 polyunsaturated fatty acids (PUFAs), such as eicosapentaenoic acid and docosahexaenoic acid, which are generated from alpha-linolenic acid. It can be found in nuts, seeds, legumes, and green leafy vegetables [124]. Omega-3 PUFAs are essential for gut epithelial integrity and serve as intra- and extracellular signaling mediators in the GI tract and brain, regulating immune regulation, inflammation, and homeostasis [125,126]. In addition, the consumption of a PUFA-enriched diet could enhance microbiome composition and diversity, as well as lead to an increase in SCFA-producing bacteria [127]. Most frequently, concurrent cognitive benefits are seen. An omega-3 PUFA diet promoted the growth of advantageous microbiota such as *Bifidobacterium* and *Lactobacillus*, improved cognition, and decreased HPA axis activity in offspring, benefits which remained until maturity when given to pregnant female C57BL/6J mice and their offspring [128]. When compared to a control diet, an omega-3 PUFA-rich diet also prevented memory decline in stressed-out adolescent male Wistar rats, normalized declines in hippocampal BDNF, and attenuated changes in the microbial composition (increased the relative abundance of *Ruminococcacea* and *Lachnospiraceae*). Even when the stressful situation had ended, these effects persisted until adulthood. Furthermore, alterations in the gut microbiota were closely related to behavioral changes. In contrast, 2 weeks of a PUFA-enriched diet resulted in substantial alterations in the microbiota composition of adolescent male Sprague Dawley rats (particularly taxa from the *Ruminococcaceae* family), but not in object or recognition memory in comparison to controls [129].

The MED diet is also rich in polyphenols, present in plant foods including vegetables, fruits, herbs, and wine, which encourage the growth of healthy bacteria in the gut [130,131]. When they are digested by the gut microbiota, the bioavailability of polyphenol-derived metabolites is increased and protects against neurotoxic harm, reduces inflammation, and supports cognitive functions [132,133]. For example, sesamol, a sesame oil-derived polyphenol, has been found to change the microbiota of mice and lessen age-related deficits. Sesamol considerably improved microbial diversity and greatly decreased cognitive impairments in old mice fed chow only, positively affecting the microbiota related to aging and inflammation. Compared to controls, oxidative stress and neuroinflammation were dramatically reduced, likely due to reduced intestinal barrier disruption and limited gut microbiome-driven LPS access into the blood [134].

### 3.3. Supplementation of Prebiotics, Probiotics, or Psychobiotics

The introduction of prebiotics, probiotics, and psychobiotics is one potential gut-targeted treatment to improve symptoms of cognitive impairment [135] and has been suggested as a possible treatment for AD [136,137]. Prebiotics are substances, generally an oligosaccharide, that are not digested but aid in beneficial bacterial growth. On the other hand, probiotics are living microbes that provide a health advantage to the host when administered in sufficient quantities. Meanwhile, psychobiotics are prebiotics or probiotics that influence the effect on the nervous system through the absorption of the substance created by the microorganisms in the intestines, which is then transported by the gut–brain axis [137,138].

After supplementation of *Morinda officinalis*, a prebiotic supplement that also contains oligosaccharides, to APP/PS1 transgenic mice, the richness of the gut microbial population is preserved, while Aβ expression, neuronal apoptosis, and brain edema are reduced [139]. A study where 3xTg-AD transgenic mice were supplemented with SLAB51 probiotics containing *Streptococcus*, *Bifidobacteria*, and lactobacilli resulted in increased levels of *Bifidobacterium* spp. and decreased levels of *Campylobacterales*, together with an increase in anti-inflammatory SCFAs and cytokines that downregulate inflammatory responses [140]. In addition, there was also a decrease in amyloid aggregation, which is associated with an improvement in cognitive function. In another study using an animal model of AD, Kobayashi and colleagues found that oral administration of *Bifidobacterium breve* strain A1 for six days in amyloid-injection-induced AD mice improves Aβ-induced cognitive deficits, where it reversed alternation behavior impairment in the Y maze test and decreased latency time in the passive avoidance test. The study was also found to reduce the expression of immune-reactive and proinflammatory genes in the hippocampus [141]. Additionally, other studies have shown that probiotics could improve cognitive deficiencies in spatial learning tests and general behavioral activities in animal models with AD [140,142,143], enhance the expression of neuronal proteolytic pathways [140], and reduce neurodegeneration [143].

In clinical studies, 12 weeks of supplementation with probiotics containing *Bifidobacterium bifidum*, *Lactobacillus fermentum*, *L. casei*, and *L. acidophilus* was found to improve cognitive performance and metabolic balance [144], while supplementation with multispecies probiotics boosted *Faecalibacterium prausnitzii* in feces and increased circulating kynurenine levels in AD patients [145]. Kynurenine is the first crucial stage in the breakdown of tryptophan by indoleamine 2,3-dioxygenase (IDO-1), where the rapid breakdown of tryptophan has been linked to cognitive deterioration in people with AD and other kinds of dementia [146]. Even though the administration of probiotics could suppress pathogen proliferation and strengthen the immune system through the intestinal barrier, combining probiotics with prebiotics improved outcomes even further [145]. Therefore, a new strategy needs to be developed to promote a healthy microbiome through the administration of psychobiotic supplements as a possible therapy for AD.

## 4. Perspectives on Diet, Lifestyle Factors, and Supplementation

**Figure 1:**
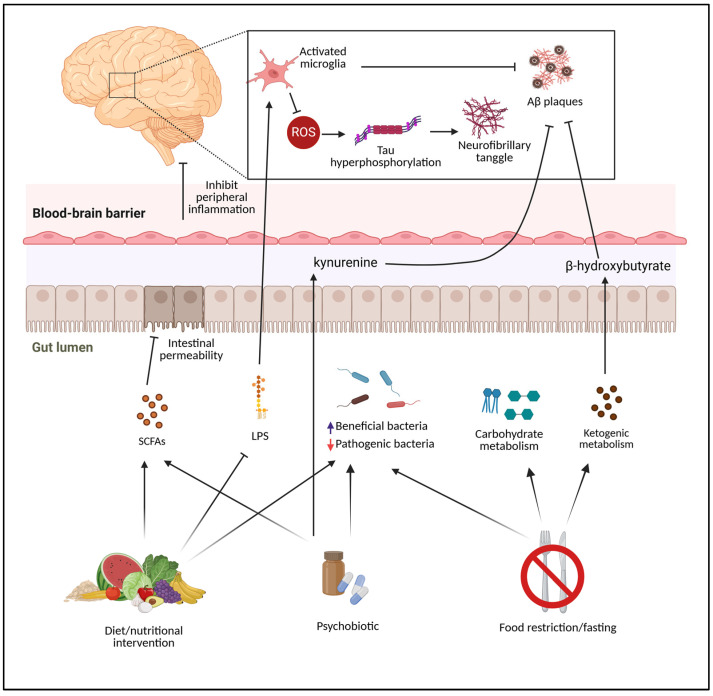
The mechanisms of the controllable factors that support the gut microbiome related to AD prevention. The growth of beneficial bacteria and SCFA-producing bacteria have the ability to maintain intestinal permeability and the production of metabolites, all of which can influence the brain and behavior via direct or indirect signaling pathways.

Healthier diets, dietary supplements, and exercise may trigger a cascade of metabolic changes in the gut microbiota that reduce inflammation and oxidative stress in AD (Figure 1). Encouraging healthy lifestyle choices among older persons to delay or prevent AD is beneficial since these lifestyle variables are easily controlled by the individual. These strategies would include healthy, nutritious diets and lifestyle behaviors.

## 5. Antioxidant Vitamins, Microbiota, and AD

**Figure 2:**
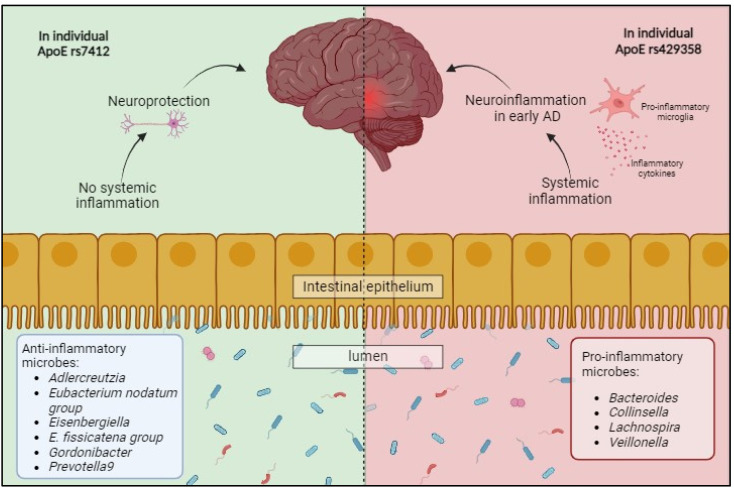
Host genetic factors present that may be modifiable by antioxidants and interactions across the microbiota–gut–brain axis (MGBA) in AD in a balanced environment between the host and microorganisms, which affects immunological and metabolic functions.

The association of oxidative stress with the development of AD has prompted the consideration of the potential protective role of antioxidants, whether obtained through dietary sources or as supplements. Oxidative stress has been reported to promote extracellular Aβ deposition and intracellular tau hyperphosphorylation [147,148]. Oxidative stress also precedes neuroinflammation in AD progression [149,150] and affects microglial function in maintaining brain health by ensuring the proper pruning of synapses. Improper pruning activity by microglia results in synaptic and neuronal loss, leading to the reduced cognitive function observed in AD [151]. Therefore, antioxidants and antioxidant vitamins, including β-carotene, vitamin E, and vitamin C, hold the potential to modulate oxidative stress and reduce the ensuing damage. Another indirect effect of antioxidant vitamins may involve reducing systemic inflammation or in situ inflammation in the gut. Increasing evidence has also pointed to the need for healthy gut microbiota to explain the presence of the gut–brain axis and its connection to the development of AD. Increasing evidence indicates a major role of gut dysbiosis and gut microbiota–host interactions in neurodegenerative diseases. Gut dysbiosis results in increased gut permeability and mediates systemic inflammation, affecting the brain barrier and leading to neuroinflammation [97]. Microbial amyloids, which mimic Aβ proteins, may be involved, triggering immune responses and resulting in microglial overactivity. Furthermore, there are interactions between host genetic factors and the gut microbiota, which affect the risk of AD [58]. In a meta-analysis study involving both discovery and replication samples, it was reported that ten genera were significantly correlated with AD. These were both protective and risk-associated genera. The genera identified as protective were primarily from the Firmicutes phylum (*Eubacterium nodatum* group, *Eisenbergiella*, and *Eubacterium fussicatena* group), Actinobacteria (*Adlercreutzia* and *Gordonibacter*), and Bacteroidetes (*Prevotella*). Those that were positively associated with risk were from phyla including Firmicutes (*Lachnospira* and *Veillonella*), Actinobacteria (*Collinsella*), and Bacteroidetes (*Bacteroides*). Of these, four genera were found to be significantly associated with the APOE rs429358 risk allele, which was in accordance with their protective/risk grouping relevant to their association with the development of AD. In contrast, the genus *Collinsella* has been consistently reported as proinflammatory and suggested to be a risk factor for AD. It was reported to be positively correlated with the APOE rs429358 risk allele in both the discovery and replication samples (Figure 2) [58]. Therefore, this raises the question of whether host genetic factors with a higher risk of developing AD may benefit from modulations to the naturally proinflammatory microenvironment in the gut through dietary changes or supplements that may support the growth of a protective microbiota and prevent or delay the onset of AD.

### 5.1. β-Carotene

Carotenoids are naturally occurring pigments present in large amounts in orange, yellow, red, and dark green fruits and vegetables. They have been suggested to play a role in preventing or delaying cognitive decline or dementia, which is possibly attributed to their antioxidant properties [152]. β-carotene, the most abundant of the carotenoids, is a powerful antioxidant and precursor for vitamin A. The antioxidant property of β-carotene is attributed to its ability to quench singlet oxygen and scavenge peroxyl radicals [153]. Due to its potent antioxidant property, β-carotene has been reported to be protective in many chronic degenerative diseases, including AD, where oxidative stress and inflammation are involved in their pathogenesis. Apart from reducing systemic oxidative stress and inflammation, it would be interesting to determine whether in situ alleviation also occurs in the gut and whether this can affect the microbiota population in the gut or through the actions of vitamin A.

A meta-analysis of antioxidant vitamins in AD patients reported that AD patients were significantly deficient in vitamin A, together with vitamin C, vitamin E, folates, and vitamin B12 [154]. Their findings reported that 333 subjects who had a diet low in vitamin A had an increased risk of getting dementia [155]. In contrast, a higher intake of β-carotene should reduce the risk of cognitive impairment, as reported by a prospective study in women in the United States [156]. The study obtained mean carotenoid intakes derived from seven repeated Food Frequency Questionnaires (FFQs) administered in 1984 and 1986 and every four years thereafter until 2006. The participants studied involved 49,493 female nurses with an average age of 48 years in 1984. Cognitive function information was self-reported using Subjective Cognitive Function (SCF) assessments in 2012 and 2014, assessed through a seven-item questionnaire on memory and cognitive function changes. The findings showed a higher intake of most commonly measured carotenoids, such as dietary β-carotene, total β-carotene, and α-carotene. Other less measured carotenoids such as lutein, lycopene, zeaxanthin, and β-cryptoxanthin were associated with substantially lower risks of having impaired or poor cognitive function. This association held true even after adjusting for other contributing factors in the diet and non-dietary risk factors, as well as considering total energy intake. In a smaller study of 68 aged subjects, individuals with AD (n = 37) exhibited lower plasma β-carotene levels compared to age-matched controls (n = 31). This difference was observed independently of other sociodemographic factors, including age, gender, smoking habits, and even genetic risk predispositions, such as the ApoE genotype. Additionally, AD patients with lower peripheral telomerase activity showed lower plasma β-carotene levels. [157]. A meta-analysis conducted by Wang et al. (2023) revealed that patients with dementia had significantly lower blood carotenoid levels when compared to controls [158]. The high heterogeneity in the reported studies added strength to this observation, although similarly low blood carotenoid levels with MCI were not observed, which was attributed to insufficient data, which warrants further investigations. Furthermore, a prospective study by Yuan et al. (2020), involving 49,493/an average of 48 SCF and FFQ tests investigating intakes of carotenoids over 22 years, showed that participants who consumed high levels of β-carotene are reported to have a lower risk of cognitive performance decline. This study was performed on a cohort of US women [156]. However, in contrast, the SUVIMAX study, which was a double-blind randomized placebo-controlled trial involving 2533 individuals aged 45–60 years old, showed that β-carotene supplementation did not decrease the risk of developing cognitive decline [159]. Other trials with single carotenoid supplements were also unable to confirm the suggested associated health benefits of carotenoids with cognitive decline. Factors that need to be considered may include doses used, possible synergistic effects with other dietary factors, and the health of the individual, their nutritional status, and their genetic background [160].

In support of the protective effect of β-carotene, an animal study using streptozotocin-induced AD in a mouse model showed that supplementation with β-carotene at a dose of 2.05/kg was found to reduce cognitive impairment and amyloid β-protein fragments, possibly via its anti-oxidative effects and the inhibition of acetylcholinesterase [161]. This raises the question of whether a diet rich in β-carotene could be protective against AD by altering inflammation in the gut, the gut microbiota, or microbiota-related metabolites, warranting further investigation.

### 5.2. Vitamin C

Vitamin C or ascorbic acid is a water-soluble vitamin that acts as a powerful antioxidant and anti-inflammatory compound in the cytosol. Apart from its antioxidant properties, other functions include synthesizing collagen and cartilage and the formation of blood vessels [162]. Several studies have reported that low plasma levels of vitamin C are associated with an increased risk of cognitive impairment, while high levels were protective [163].

The antioxidant and anti-inflammatory properties of vitamin C naturally link it to AD pathogenesis, although definitive evidence in humans will require interventions via costly randomized controlled trials. Biological evidence linking diets high in vitamin C with the risk of AD is increasing, which is of interest as modifying diets can be a smart lifestyle choice. It is then possible that dietary interventions may lead to epigenetic modifications of the human genome, leading to the prevention of AD and possibly delaying the development of AD in susceptible individuals.

A study of 79 patients with AD measuring several antioxidants together with vitamin C showed that total antioxidant capacity (TAC) was significantly reduced in AD as well as other dementia groups such as vascular dementia (VaD) and Parkinson’s disease (PD). TAC measures plasma carotenoids (α carotene, β carotene, and lycopene), vitamin A, vitamin C, and vitamin E. Determinations of individual antioxidants revealed that itamins A, C, and E were reduced in both AD and VaD patients compared to controls. In addition, β carotene was low in VaD patients, while PD patients had reduced lycopene levels. The reduction in plasma antioxidants in patients with dementia may indicate the presence of high free radical activity, leading to oxidative stress and inflammation, which are known pathologies for the different types of dementia [164].

Vitamin C is neuroprotective through mechanisms that include general free radical trapping, suppression of pro-inflammatory genes, reducing neuroinflammation, and preventing the chelation of metals associated with free radical production, including iron, copper, and zinc. The formation of Aβ fibrillogenesis is then mitigated, which is an early step in the development of neurodegeneration preceding cognitive decline [165]. In animal experiments, vitamin C deficiency has been shown to increase oxidative stress, leading to impaired amyloid accumulation and deposition and impairing cognition [166]. Furthermore, vitamin C supplementation in APP/PSEN1 mice has been shown to reduce Aβ oligomerization, resulting in a reduction in spatial learning without affecting plaque formation [167]. Another study has shown that homocysteine levels are raised, which has been known to promote DNA damage, thus sensitizing neurons to Aβ toxicity [168].

In animal studies, both in vivo and in vitro, treatment with vitamin C (ascorbic acid) and vitamin E (D-alfa-tocopherol acetate) either individually or in combination have shown to decrease oxidative DNA damage, suggesting the ability of these two antioxidant vitamins to mitigate the development of oxidative stress [169]. A study found evidence that genetic liability to higher vitamin C levels was associated with a lower risk of AD (OR ¼ 0.968; 95% CI) and cardioembolic stroke (odds ratio ¼ 0.773; 95% confidence interval (CI)) [170]. However, studies linking vitamin C with the gut microbiota and AD are largely unexplored.

### 5.3. Vitamin E

The debate continues on the possible protective role of vitamin E in preventing AD through its function as a lipid-soluble antioxidant, protecting cells against harmful free radicals, reducing oxidative stress and inflammation, and enhancing the immune response in elderly people, especially through its α-tocopherol form [171]. Furthermore, vitamin E exists in two main isomer forms, tocopherols and tocotrienols, each with α, β, δ, and γ isomers depending on the position of CH3 groups [172]. Observational studies have reported low circulating plasma levels of α-tocopherol in AD patients compared to normal individuals in several small human studies [164,173], while high levels were protective in delaying AD [174,175]. A total of three meta-analysis studies have reported that AD patients and people with age-related cognitive decline had circulating concentrations of vitamin E that were significantly lower than the healthy control group [154,176,177]. However, the findings from a prospective observational study involving 980 subjects, which assessed vitamin C and E intake over a four-year period, did not reveal any significant difference in AD incidence when followed up over a period of 4 years [169].

A prospective supplementation study involving 4740 participants reported a reduction in AD prevalence (OR 0.22; 95% CI 0.05 to 0.60) and incidence (HR 0.36; 95% CI 0.09 to 0.99) when both vitamin E (400 IU) and vitamin C (500 mg) were taken daily over a period of at least 3 years. Several clinical trials have demonstrated the protective effects of vitamin E in relation to the development of AD. In a randomized placebo trial called the TEAM-AD VA trial, which involved patients with mild to moderate AD, the progression of cognitive impairment was significantly delayed with supplementation of 2000 IU/d α-tocopherol over a period of two years [178]. Another placebo-controlled, randomized, and double-blind multicenter trial, also involving patients with moderate AD, observed that supplementation with α-tocopherol (2000 IU/d) for two years significantly delayed disease progression when assessed by CDR questionnaires and their ability to perform basic daily living activities [179]. The above observational, interventional, and meta-analysis findings are from just some of the reported studies that showed the potential protective properties of vitamin E in the development of AD. It should be noted that many more studies are available that link vitamin E with AD.

In contrast, an intervention study, the PREADVISE study, where α-tocopherol was supplemented either alone or with selenium to 7640 elderly men for an average of 5.4 years, also did not show any difference in dementia incidence [180]. Responses to vitamin E supplementation have been variable and more complex to decipher, as the protective effect of vitamin E that is consistently observed in animal and in vitro studies has not been fully translated into human intervention trials. It has been suggested that this may be due to differences in doses used, duration of supplementation, genetic makeup, nutritional status of the participants, type of vitamin E used, and severity of AD at the start of intervention. Based on the mechanism of action of vitamin E as an antioxidant and signaling molecule, interventions would be expected to be effective in nutritionally compromised individuals, mildly cognitively impaired individuals, or moderate AD patients [181,182,183,184].

As mentioned previously, human studies relating to vitamin E and AD have yielded inconsistent findings. However, AD animal model studies have provided fairly strong evidence of the potential protective effect of vitamin E on the development of AD. Several animal studies using transgenic mice, such as the APP1/PS1 study, have shown that supplementation with vitamin E had a protective effect in delaying the onset of AD symptoms [185,186,187,188,189].

The presence of the gut–brain axis and increasing evidence showing gut microbiota changes in AD patients raise the question of how antioxidants affect the microenvironment and influence the diversity of the microbiota population in the gut. However, very few studies are available to relate the antioxidant properties of vitamin E with changes in the gut microbiota in either animal models or human studies, which warrants further investigations to ascertain the potential of vitamin E as a fairly simple therapeutic preventive strategy.

## 6. Future Perspectives

An individual’s gut microbiota is influenced by genetics, as shown by twin studies [190]. However, environmental factors including diet, drugs, and anthropometric measures have a larger effect in determining a person’s microbiota composition [191]. There is increasing evidence of the modifiability of the gut microbiota through dietary components, which may include prebiotics, probiotics, fiber, and carbohydrates, particularly refined sugar, thus impacting health and disease [192,193]. In addition, the basis for the effect of nutraceuticals is mainly from fruits and vegetables, i.e., food or food products that provide medical or health benefits, including preventing and treating diseases in AD through protection against mitochondrial damage, oxidative stress, inflammation, and the toxicity of Aβ and tau proteins [194,195,196].

The gut microbiota is molded by diet, which then affects the host’s metabolism and determines the health status of the individual, as reported previously by Asnicar et al., 2021 [197]. In the Personalised Responses to Dietary Composition Trial (PREDICT 1) study, 1098 participants contributed 1203 gut microbiomes, which were then subjected to deep metagenomic sequencing. The study revealed numerous significant associations between the type of microbes and intake of specific nutrients, types of foods, different food groups, and even overall dietary indices. The presence of different types of healthy and plant-based foods notably influenced these associations. Although the search continues to identify the key composition of a healthy ‘microbiota’, gut microbiota dysbiosis has been reported in diseases, including AD, which offers a potential therapeutic target to prevent or delay the onset of AD. For example, in AD mouse models, alterations in gut microbiota composition result in the peripheral accumulation of branch chain amino acids, such as phenylalanine and isoleucine, stimulating the differentiation and proliferation of pro-inflammatory T helper 1 cells, which then infiltrate the brain, resulting in microglia activation and neuroinflammation, which are early events in the development of AD [198]. These amino acids and T helper 1 cells were also elevated in patients with mild cognitive impairments. Interestingly, the authors continued the intervention by using sodium oligomannate, which restored gut eubiosis with concomitant reduced phenylalanine and isoleucine accumulation and neuroinflammation, along with solid and consistent cognition improvement. These findings highlight the role of gut-promoted neuroinflammation and suggest a new therapeutic target for AD therapy by remodeling the gut microbiota. Thus, the complex interactions and relationship between the diet–gut microbiota–brain axis and AD and the mechanisms involved warrant further investigation, particularly involving antioxidant vitamins and supplements. These may include microbial metabolites such as SCFAs, immune responses, systemic inflammation and neuroinflammation, and host gene–microbiota interactions.

## 7. Conclusions

Overall, there is increasing evidence to support the effect of the microbiota on the development of AD through the gut microbiota–brain axis. Human studies have reported on the presence of gut dysbiosis and the presence of pro-inflammatory species such as *Collinsella and Veillonella* [58] in AD. Furthermore, GWAS studies have shown the presence of host genetic interactions, particularly ApoE4 with pro-inflammatory species. A limited number of animal model studies have shown that the remodeling of gut microbiota by antioxidant compounds has been able to reduce the development of cognitive decline in transgenic animals. However, both human and animal studies on the modeling of the gut microbiota by antioxidant vitamins and the development of AD are fairly limited, particularly when nutraceuticals in the diet have been reported to modulate the gut microbiome [199]. Currently, clinical trials using FMT are ongoing to determine if there will be an improved response to chemotherapy; however, this is for other diseases and not AD. This may warrant future studies considering the lack of effective treatments for AD.

## Figures and Tables

**Table 1:** Global studies on amyloid beta-producing gut bacteria in AD.

Studies	Countries	Study Design	Participants	Comments
Ferreiro et al. (2023) [57]	USA	Cross-sectional study	164 cognitively normal individuals and 49 early preclinical AD patients	The composition of gut microbiota in individuals displaying preclinical AD (characterized by changes in brain amyloid and Tau proteins) differed from that of healthy individuals.
Cammann et al. (2023) [58]	USA	A combination of cross-sectional studies	MiBioGen consortium: (ADc12 case/control: 1278/1293) and (GenADA case/control: 799/778)	The study identified several gut microbiota that were associated with AD. The protective factors included the *Eubacterium nodatum* group, *Eisenbergiella*, the *Eubacterium fissicatena* group, *Adlercreutzia*, *Gordonibacter*, and *Prevotella*. The risk factors included *Lachnospira*, *Veillonella*, *Collinsella*, and *Bacteroides*. These genera were found to have a significant correlation with AD diagnosis and the APOE genotype.
Wanapaisan et al. (2023) [59]	Thailand	Cross-sectional study	normal; *n* = 20, MCI; *n* = 12, and AD; *n* = 20	A significantly higher abundance of bacteria in nondementia patients belonged to the *Clostridiales* order, including *Clostridium sensu stricto* 1, *Fusicatenibacter*, *Lachnospiraceae*, *Agathobacter*, and *Fecalibacterium*. In contrast, *Escherichia*-*Shigella*, *Bacteroides*, *Holdemanella*, *Romboutsia*, and *Megamonas* were the dominant genera in the AD group.
Dilmore et al. (2023) [60]	USA	Clinical trial study	Control = 11; MCI = 9	A low-fat diet was associated with lower levels of GABA-producing microbes in individuals with MCI.
Xia et. al. (2023) [61]	USA	Post Hoc Analysis based on a clinical trial study	66 participants	There was a significant increase in family and genus alpha-diversity in both groups combined, but no other significant changes in gut microbiota structure, abundance, or function due to the effects of a Med Diet.
Ying et al. (2023) [62]	China	Cross-sectional study	118 subjects (45 AD, 38 MCI, and 35 NC)	The study found that patients with cognitive impairments had altered gut microbiota, with a lower abundance of *Dorea* and higher levels of DAO, DA, and ET. The study also predicted functional changes in gut microbiota pathways, showing alterations in glycan biosynthesis and metabolism in MCI patients. Additionally, the abundance of *Bacteroides* and *Faecalibacterium* was negatively correlated with ET levels and positively correlated with cognitive function scores.

**Table 2:** Impact of antioxidant interventions on gut microbiota in animal studies.

Studies	Countries	Study Design	Participants	Comments
Nagarajan et al. (2023) [63]	USA	Case-control study	Not specified, using AD rat model; Tgf344-AD WT control rats at various ages	In the Tgf344AD rats, changes were evident in genera such as *Bifidobacterium*, *Ruminococcus*, *Parasutterella*, *Lachnoclostridium*, and *Butyricicoccus*. Age-related shifts observed in both the Tgf344-AD and WT control rats included reductions in *Enterohaldus*, *Escherichia*, *Shigella* and *Rothia*, and increases in *Turicibacter* and *Clostrium*_*senso*_*stricto*.
Jin et al. (2023) [64]	China	In vivo study	AD mouse model; 14 WT mice (C57BL/6) and 16 APP/PS1 mice	The study observed that gut Aβ42, a more toxic form of Aβ, increased with age in wild-type and mutant APP/PS1 mice. Furthermore, the alterations in gut microbiota composition in aged APP/PS1 mice and the impact of FMT on gut Aβ production were investigated.
Borsom et. al. (2023) [65]	USA	A longitudinal study	AD mouse model; 3xTg-AD mice: 39 Wild-type (WT) mice: 24	The study found that the gut microbiota of 3xTg-AD mice was compositionally distinct from WT mice early in life. Bacterial features, such as *Akkermansia muciniphila*, *Turicibacter* species, *Prevotella* species, *Bacteroides acidifaciens*, and *Lactobacillus salivarius*, were differentially enriched in 3xTg-AD mice over time.
Jun Fu et. al. (2023) [66]	China	A pre-clinical study	AD rat model; 4 groups (*n* = 6) including a sham-operated group (Sham), a model group (AD), an SCP-treated group (SCP), and an SCP2-treated group (SCP2).	Fecal metabolomic and microbiome analyses revealed that SCP2 modulated metabolites and reversed gut microbiota disorders in AD rats. Additionally, SCP2 increased the content of SCFAs in the AD rats. SCP2 had significant therapeutic effects in AD rats, improving learning and memory capacity, reducing neuroinflammation, and restoring the integrity of the intestinal barrier.
Guan et al. (2023) [67]	China	A pre-clinical study	AD mouse model; 8 = WT, 8 = APP/PS1, and 8 = HC-treated APP/PS1)	HC demonstrated neuroprotective effects in Alzheimer’s disease by reducing Aβ and p-tau levels, modulating gut microbiota composition, and promoting beneficial bacteria. It also decreased D-glutamic acid and oxidized glutathione levels, reduced ROS, and enhanced antioxidant enzyme secretion, highlighting its role in regulating oxidative stress and the gut microbiota.
Yang et al. (2023) [68]	China	A pre-clinical study	Not specified, using a mouse model	The study demonstrated that nanoparticles effectively delivered Res to the brain, improving cognitive function in obesity-related Alzheimer’s disease mice. They also alleviated insulin resistance, reduced Tau phosphorylation and Aβ aggregation, and regulated glucose homeostasis, oxidative stress, and neuroinflammation. The nanoparticles modulated gut microbiota associated with inflammation, lipid metabolism, and oxidative stress, including species such as *Helicobacter*, *Bifidobacterium*, and *Candidatus_Saccharimonas.*
Cuervo-Zanatta et al. (2022) [69]	Mexico	A pre-clinical study	AD mouse model; WT-C *n* = 10, Tg-C *n* = 10, Tg-F *n* = 10, and Tg-F-Abx *n* = 10	Soluble fiber intake can modulate gut bacteria to increase butyrate and decrease propionate levels, improving the composition of gut microbiota. This modulation reduced astrocyte activation and enhanced cognitive function in an AD mouse model, suggesting that dietary interventions rich in soluble fiber may help prevent cognitive decline and AD.
Qiao et al. (2022) [70]	China	A pre-clinical study	AD mouse model; 40 male C57BL 6 mice aged 8 weeks.	The study explored the protective effects of *L. casei* ATCC 393-SeNPs on cognitive dysfunction in an AD mouse model. The treatment improved cognitive function, reduced Aβ aggregation and tau hyperphosphorylation, and prevented neuronal death. It also restored intestinal barrier function, balanced gut microbiota, and increased short-chain fatty acids and neurotransmitter levels.
Zhang et al. (2022) [71]	Korea	A pre-clinical study	AD rat model; 50 Aβ (25–35)-infused rats divided into five groups, with 10 rats in each group	The study found that low-molecular-weight fucoidan and λ-carrageenan improved memory function in the rats. Additionally, the study found that the supplementation improved glucose metabolism and modulated the composition of the gut microbiota, potentially contributing to the observed cognitive improvements. AD-Con increased *Clostridium*, *Terrisporobacter*, and *Sporofaciens* compared to Normal-Con, and AD-F-L and AD-C-L increased *Akkermentia*.
Yang et al. (2022) [72]	China	A pre-clinical study	AD mouse model; 25 mice (5 from each group)	The results regarding the nanoparticles’ antioxidant properties showed that they exhibited DPPH- and ABTS+-scavenging activity, indicating their ability to scavenge free radicals. Specifically, the nanoparticles influenced the abundance of *Firmicutes*, *Bacteroidota*, *Desulfobacterota*, *Actinobacteriota*, and *Patescibacteria*. These findings suggest that the nanoparticles can regulate the composition of the gut microbiota.
Zhang et al. (2022) [73]	China	A pre-clinical study	Not specified, using AD mouse model	EMR mice showed metabolic disorders, gut microbiota dysbiosis, inflammation, and AD-like symptoms. FMT improved gut permeability, reduced inflammation, and alleviated AD-related phenotypes and obesity. The study suggests that EMR-induced gut microbiota dysbiosis contributes to AD and obesity, making the gut microbiota a potential target for prevention and treatment. Correlation analysis indicated that systemic inflammation might be linked to the increased abundance of *Ruminiclostridium*.
Cox et al. (2022) [74]	USA and France	A pre-clinical study	AD mouse model; 11–13 for SOD1 mice (Abx), 11–13 for SOD1 mice (CoHo), and 11–13 for untreated SOD1 mice (H2O).	The study found that low-dose antibiotic treatment worsened disease progression in mice by reducing beneficial bacteria and increasing neurodegenerative genes in microglia. Co-housing with wild-type mice had no significant effect. The results suggest that the microbiota plays a protective role in ALS by restraining neurodegenerative microglia. Antibiotics reduced *Akkermansia* and butyrate-producing bacteria, which may be beneficial in ALS, while co-housing had a minimal impact on the microbiome.
Li et al. (2021) [75]	China	A pre-clinical study	Not specified, using a mouse model	The nanocomposite TGN-Res@SeNPs improved cognitive function in mice by reducing Aβ aggregation in the brain, decreasing oxidative stress, and enhancing antioxidant enzyme activity. It also suppressed Aβ-induced neuroinflammation through the NF-kappa B/MAP kinase/Akt signaling pathway. Additionally, TGN-Res@SeNPs improved gut microbiota balance by reducing inflammation-related bacteria, such as *Alistpes*, *Helicobacter*, *Rikenella*, *Desulfovibrio*, and *Faecalibaculum.*
Borah et al. (2022) [76]	India	A pre-clinical study	AD rat model; A total of 18 female Wistar Albino rats were divided into three groups, each consisting of six rats.	Analysis of fecal microbiota revealed a shift from the *Bacteroides* genus to the *Lactobacillus* genus in the treated group compared to the demented group. Zederone, a compound with a purity of 96.57%, caused a reduction in the accumulation of amyloid plaques, as observed under a fluorescence microscope.
Xu et al. (2021) [77]	China	A pre-clinical study	A mouse model using 30 male mice. The grouping was not specified	Q3G reversed dysbiosis of the gut microbiota and the decrease in short-chain fatty acids caused by A β 1-42. Quercetin-3-O-Glucuronide alleviates cognitive deficits and toxicity in Aβ_1-42_-induced AD-like mice and SH-SY5Y cells.
Liu et al. (2020) [78]	China	A pre-clinical study	AD mouse model; 24 APP/PS1 transgenic mice and 12 wild-type mice as the control group	Ingestion of AR-C17 dramatically altered gut dysbiosis in APP/PS1 transgenic mice by increasing the relative abundance of *Lactobacillus* and *Akkermansia* and decreasing the abundance of *Clostridium* and *Desulfovibrio*.
Weng et al. (2020) [79]	Taiwan	A preclinical study	Not specified, using an AD rat model	Camellia oil prevents the development of AD by reducing memory deficits, improving learning capacity, boosting antioxidant activity, controlling the expression of immune-related cytokines, promoting autophagy, and improving the composition of gut microbiota in aluminum chloride-treated rats. Camellia oil can reverse AD brain pathology.
Shen et al. (2019) [80]	China	A pre-clinical study	AD mouse model; A total of 24 transgenic APP/PS1 mice were divided randomly into three groups.	Silibinin and silymarin were able to improve memory deficiencies and led to less amyloid plaque buildup than in controls. Additionally, silibinin and silymarin administration had a tendency to reduce the diversity of the microbiota. They had a regulating influence on the abundance of numerous important bacterial species linked to AD development.

## Data Availability

Data are contained within the article.
